# Supplementary material for: Analyses of antioxidant status and nucleotide alterations in genes encoding antioxidant enzymes in patients with benign and malignant thyroid disorders
Source: PeerJ. 2017 Jun 1;5:e3365. doi: 10.7717/peerj.3365 (PMC5457668; doi:10.7717/peerj.3365)
Supplement: Supplemental Information 1 [file peerj-05-3365-s001.pdf]

Antioxidant activities in serum

SOD

n= x	Groups	Average	SEM
14	CONTROL	3.123552	0.370493
18	MNG	14.74056	2.018678
10	PTC	15.07055	3.112137
7	FTA	11.3415	2.685847
6	FTC	16.25202	6.246038

CAT

n= x	Groups	Average	SEM
14	CONTROL	67.8876	6.995937
18	MNG	162.8241	28.45832
10	PTC	210.6742	42.89895
7	FTA	158.0128	65.68318
6	FTC	246.2139	83.45263

GPX

n= x	Groups	Average	SEM
14	CONTROL	122.8584	15.48552
18	MNG	92.06669	12.29061
10	PTC	128.4495	14.00393
7	FTA	156.0654	9.440131
6	FTC	114.6112	26.52104

ABTS

n= x	Groups	Average	SEM
14	CONTROL	1.629282	0.023779
18	MNG	0.982575	0.057909
10	PTC	0.594068	0.033374
7	FTA	1.304267	0.033681
6	FTC	1.345461	0.030763

Lipid Peroxidation

n=x	Groups	Average	SEM
14	CONTROL	0.016368	0.00054
18	MNG	0.018637	0.000769
10	PTC	0.018633	0.0006
7	FTA	0.020935	0.000651
6	FTC	0.022167	0.002555

ROS

n= x	Groups	Average	SEM
14	CONTROL	90.61905	4.517887
18	MNG	107.9259	5.358028
10	PTC	131.2667	9.901715
7	FTA	102.2857	5.959488
6	FTC	87.77778	7.586375
