# Supplementary material for: Analyses of antioxidant status and nucleotide alterations in genes encoding antioxidant enzymes in patients with benign and malignant thyroid disorders
Source: PeerJ. 2017 Jun 1;5:e3365. doi: 10.7717/peerj.3365 (PMC5457668; doi:10.7717/peerj.3365)
Supplement: Supplemental Information 2 [file peerj-05-3365-s002.pdf]

Antioxidant activities in RBC

SOD

n=x	Groups	Average	SEM
14	CONTROL	772.6549	19.88351
18	MNG	651.7279	25.78951
10	PTC	674.6361	19.75752
7	FTA	670.1492	32.19458
6	FTC	650.8587	27.46191

CAT

n=x	Groups	Average	SEM
14	CONTROL	40121.46	4856.581
18	MNG	35038.04	4705.932
10	PTC	17883.57	7660.464
7	FTA	10567.3	5557.09
6	FTC	27565.46	5682.723

GPX

n=x	Groups	Average	SEM
14	CONTROL	1357.143	62.62929
18	MNG	1219.832	88.88595
10	PTC	75.64343	23.738
7	FTA	1443.01	80.88666
6	FTC	197.8105	103.7394

ABTS

n=x	Groups	Average	SEM
14	CONTROL	1.667459	0.132068
18	MNG	1.095622	0.136948
10	PTC	1.36436	0.251316
7	FTA	1.583176	0.23635
6	FTC	1.057226	0.307984

Lipid Peroxidation

n=x	Groups	MEAN	SEM
14	CONTROL	0.019245	0.000563
18	MNG	0.027441	0.000721
10	PTC	0.024898	0.00126
7	FTA	0.018452	0.000728
6	FTC	0.019178	0.001087

ROS

n= x	Groups	Average	SEM
14	CONTROL	1291.607	30.98388
18	MNG	1372.167	77.83841
10	PTC	1640.3	77.58375
7	FTA	997.5714	100.9601
6	FTC	1362.083	224.6239
