# Supplementary material for: Analyses of antioxidant status and nucleotide alterations in genes encoding antioxidant enzymes in patients with benign and malignant thyroid disorders
Source: PeerJ. 2017 Jun 1;5:e3365. doi: 10.7717/peerj.3365 (PMC5457668; doi:10.7717/peerj.3365)
Supplement: Supplemental Information 3 [file peerj-05-3365-s003.pdf]

Results of Whole Exome Sequencing in MNG and PTC patients

MNG n=9	Ge					
	SOD1	*SOD2*	*SOD3*	*GPX1*	*GPX2*	*GPX3*
	S					
**B1**		rs4880<br>rs752779	rs8192291<br>rs1799895<br>rs2695232<br>rs2855262			rs11548<br>rs2070593<br>rs2277940
**B2**		rs752779	rs8192291<br>rs2695232<br>rs2855262			rs11548<br>rs2277940
**B3**		rs752779	rs8192291			rs11548<br>rs2070593<br>rs2277940
**B4**			rs8192291			
**B5**		rs752779	rs2536512<br>rs2695232<br>rs2855262			rs870407<br>rs3763012<br>rs3763011<br>rs869976<br>rs8177447<br>rs11548<br>rs2070593<br>rs2277940
**B6**		rs4880<br>rs2855116<br>rs2070994<br>rs2842960<br>rs752779	rs2695232<br>rs2855262			rs8177412<br>rs870407<br>rs870406<br>rs3763012<br>rs3763011<br>rs869975<br>rs8177447<br>rs11548<br>rs2277940
**B7**		rs4880<br>rs2758332<br>rs2855116<br>rs2070994<br>rs2842960<br>rs752779				rs8177412<br>rs870407<br>rs870406<br>rs3763011<br>rs8177447<br>rs2070593
**B8**		rs4880<br>rs2855116<br>rs2070994<br>rs2842960<br>rs752779	rs2536512<br>rs8192291<br>rs2695232<br>rs2855262			rs870407<br>rs870406<br>rs3763011<br>rs8177447
**B9**		rs4880<br>rs2758332<br>rs2855116<br>rs2070994<br>rs2842960<br>rs752779	rs2536512<br>rs2695232<br>rs2855262			rs8177447

enes					
GPX4	*GPX5*	*GPX6*	*GPX7*	*GPX8*	*CAT*
NPs					
			rs1970951	rs381852	rs769217
rs713041			rs1970951	rs381852<br>rs10059517	rs704724<br>rs7943316<br>rs1049982
rs713041			rs1970951<br>rs11810754	rs381852	rs7943316
rs4807542<br>rs713041			rs1970951	rs381852	
rs713041<br>rs8178977		rs974334	rs1970951	rs381852	rs769217<br>rs10836235<br>rs769218<br>rs16925614<br>rs17881488
			rs1970951		rs7943316<br>rs769217<br>rs1049982<br>rs10836235<br>rs769218<br>rs17881488
rs4807542<br>rs713041			rs1970951	rs381852<br>rs2270910<br>rs10059517	rs769217<br>rs769218<br>rs16925614
			rs1970951	rs381852	rs7943316<br>rs1049982<br>rs12270780<br>rs2073058
rs713041			rs1970951	rs381852	rs7943316<br>rs1049982

PTC<br><br>n=9	[illegible]					
	SOD1	*SOD2*	*SOD3*	*GPX1*	*GPX2*	*GPX3*
	[illegible]					
**M1**		rs4880<br>rs752779	rs2536512<br>rs2695232<br>rs2855262		rs17881414	
**M2**		rs752779	rs8192291<br>rs2695232<br>rs2855262	rs368623389		rs11548<br>rs2277940
**M3**		rs752779	rs2536512<br>rs8192291<br>rs2695232<br>rs2855262			rs8177413<br>rs11548<br>rs2230303<br>rs8177448<br>rs2070593<br>rs2277940
**M4**		rs752779	rs8192291			rs8177412
**M5**		rs4880<br>rs2758332<br>rs2855116<br>rs2842960<br>rs752779	rs2536512<br>rs2695232<br>rs2855262			rs3763011<br>rs869975<br>rs8177447<br>rs11548<br>rs2070593<br>rs2277940
**M6**		rs4880<br>rs2758332<br>rs2855116<br>rs2070994<br>rs2842960<br>rs752779	rs2536512<br>rs2695232<br>rs2855262			rs8177413<br>rs870407<br>rs3763012<br>rs3763011<br>rs869976<br>rs8177447<br>rs2230303<br>rs2277940
**M7**		rs4880<br>rs2758332<br>rs2855116<br>rs2070994<br>rs2842960<br>rs752779	rs2536512<br>rs2695232<br>rs2855262			rs3763011<br>rs869975<br>rs8177447<br>rs11548<br>rs2277940
**M8**						rs8177412<br>rs870407<br>rs870406<br>rs3763011<br>rs8177447<br>rs2070593
**M9**		rs752779	rs2536512<br>rs8192291			rs8177447

Genes					
GPX4	***GPX5***	***GPX6***	***GPX7***	***GPX8***	***CAT***
**SNPs**					
rs713041		rs372898956	rs1970951	rs381852	rs769217
			rs1970951	rs381852	rs769217<br>rs7943316<br>rs1049982
rs569667691			rs1970951<br>rs11810754	rs381852<br>rs10059517	rs769217
rs713041			rs1970951<br>rs11810754	rs381852<br>rs10059517	rs769217
		rs974334	rs1970951	rs381852	rs7943316<br>rs1049982<br>rs12270780<br>rs2073058
			rs1970951<br>rs11810754	rs381852<br>rs2270910<br>rs10059517	
rs713041		rs974334	rs1970951	rs381852	rs769217<br>rs10836235<br>rs769218<br>rs16925614
			rs1970951		rs769218<br>rs769217
rs8178977		rs372898956<br>rs974334	rs1970951<br>rs11810754	rs381852	rs7943316<br>rs769217<br>rs1049982<br>rs769218
